# Bioengineering strategies for next-generation female reproductive organoids: A roadmap to clinical translation

**DOI:** 10.1063/5.0332917

**Published:** 2026-10-26

**Authors:** Bowen Gao, Jianing Gao, Wenbo Wei, Yixuan Hu, Chengran Hu, Wentao Su, Shuo Chen, Li Wang

**Affiliations:** 1 Roger Williams Institute of Liver Studies, School of Immunology & Microbial Sciences, Faculty of Life Sciences & Medicine, King's College London, United Kingdom; 2 State Key Laboratory of Marine Food Processing and Safety Control, Academy of Food Interdisciplinary Science, School of Food Science and Technology, Dalian Polytechnic University, Dalian, Liaoning, China; 3 R&D Department, Suzhou Jiyan Biopharmaceutical Technology, Taicang Biomedical Industrial Park, Suzhou, China; 4 Department of Gynecology, The Third Affiliated Hospital of Guangzhou Medical University, Guangzhou, Guangdong, China

## Abstract

The human female reproductive system is characterized by highly dynamic tissue remodeling, cyclical endocrinology, and the precise orchestration of maternal–fetal immune tolerance. Historically, deciphering reproductive pathologies ranging from infertility to obstetric complications and malignancies has been hampered by the fundamental physiological and interspecies disparities inherent in 2D cultures and animal models. While three-dimensional (3D) organoid technology has initiated a paradigm shift, conventional static cultures relying on poorly defined, xenogeneic matrices (e.g., Matrigel) introduce profound immunogenic confounding factors and severe bioengineering bottlenecks. To bridge this gap, this review comprehensively delineates the advanced bioengineering strategies driving the next-generation of female reproductive models. We first outline foundational engineering pillars, highlighting the transition toward intelligent biomaterials, deterministic 3D bioprinting, and microfluidic microenvironments. Subsequently, we critically evaluate state-of-the-art organoid models across the ovary, fallopian tube, the maternal–fetal interface containing endometrium and placenta, cervix, and vagina. We emphasize how multi-lineage “assembloids” and multiorgan microphysiological systems successfully recapitulate transient implantation events and bidirectional endocrine crosstalk. Finally, we propose a strategic roadmap for the field, demonstrating how the integration of materials science, genetics, multi-omics, and engineering methods will overcome current limitations, ultimately accelerating the transition from benchtop *in vitro* models to standardized clinical diagnostics and reproductive regenerative therapeutics.

## I. INTRODUCTION

The human female reproductive tract is a masterclass in dynamic physiological remodeling, characterized by intricate cyclical endocrinology, profound mucosal barrier regeneration, and the orchestration of precise maternal–fetal immune tolerance. Consequently, dysregulation within this highly specialized system leads to a wide spectrum of reproductive pathologies, ranging from infertility, endometriosis, and recurrent pregnancy loss to lethal malignancies such as high-grade serous ovarian carcinoma (HGSC). Historically, deciphering the pathophysiology of these complex diseases has been severely hampered by the fundamental inadequacies of conventional preclinical models. Standard 2D monolayer cultures could not satisfactorily recapitulate the 3D cytoarchitecture and mechanotransduction cues inherent to native tissues.^1–4^ In addition, interspecies variations such as the divergent 4–5 day murine estrous cycle^5^ vs the 28-day human menstrual cycle, alongside critical immunological differences in human hemochorial placentation,^6–8^ severely limit the clinical translatability of animal-derived data.

The advent of three-dimensional (3D) organoid technology has initiated a paradigm shift in reproductive biology. Derived from adult stem cells (ASCs) or pluripotent stem cells (PSCs),^9^ these self-organizing microtissues successfully replicate the foundational epithelial architecture, genetic signatures, and basic hormonal responsiveness of native reproductive organs.^10,11^ However, as the field reshapes from basic developmental biology to precision disease modeling and regenerative medicine, conventional organoid cultures face critical bioengineering bottlenecks. The heavy reliance on poorly defined, xenogeneic matrices (e.g., Matrigel) introduces profound immunogenic confounding factors and batch-to-batch variability.^12^ Moreover, current models often function as reductionist factor-isolated systems that are inherently incapable of modeling the dynamic fluidic shear stress, precise spatiotemporal organization, and systemic multi-organ endocrine crosstalk that dictate female reproductive physiology. To bridge the chasm between reductionist *in vitro* cultures and complex *in vivo* microenvironments, a transition toward holistic physiological integration is urgently required.

This review aims to comprehensively delineate the bioengineering strategies driving the next-generation of female reproductive models. We first deliver a comprehensive overview of the core engineering elements including biomaterials, cellular components, biofabrication, and microfluidics. Subsequently, we critically evaluate the state-of-the-art organoid models for each reproductive organ, emphasizing how physiological dynamics and bidirectional crosstalk are recapitulated through advanced engineering. Finally, we propose a strategic roadmap, identifying current limitations and emerging strategies that will catalyze a paradigm shift toward precision reproductive medicine.

## II. OVERVIEW: ENGINEERING PRINCIPLES FOR FEMALE REPRODUCTIVE ORGANOIDS

Engineering female reproductive organoids requires a multi-disciplinary approach to replicate the unique cyclicity and dynamic remodeling characteristic of these tissues. This overview outlines the core engineering pillars: biomaterial selection, cellular sources, assembly strategies, and the technological platforms used to control the microenvironment (Fig. 1).

**FIG. 1.**
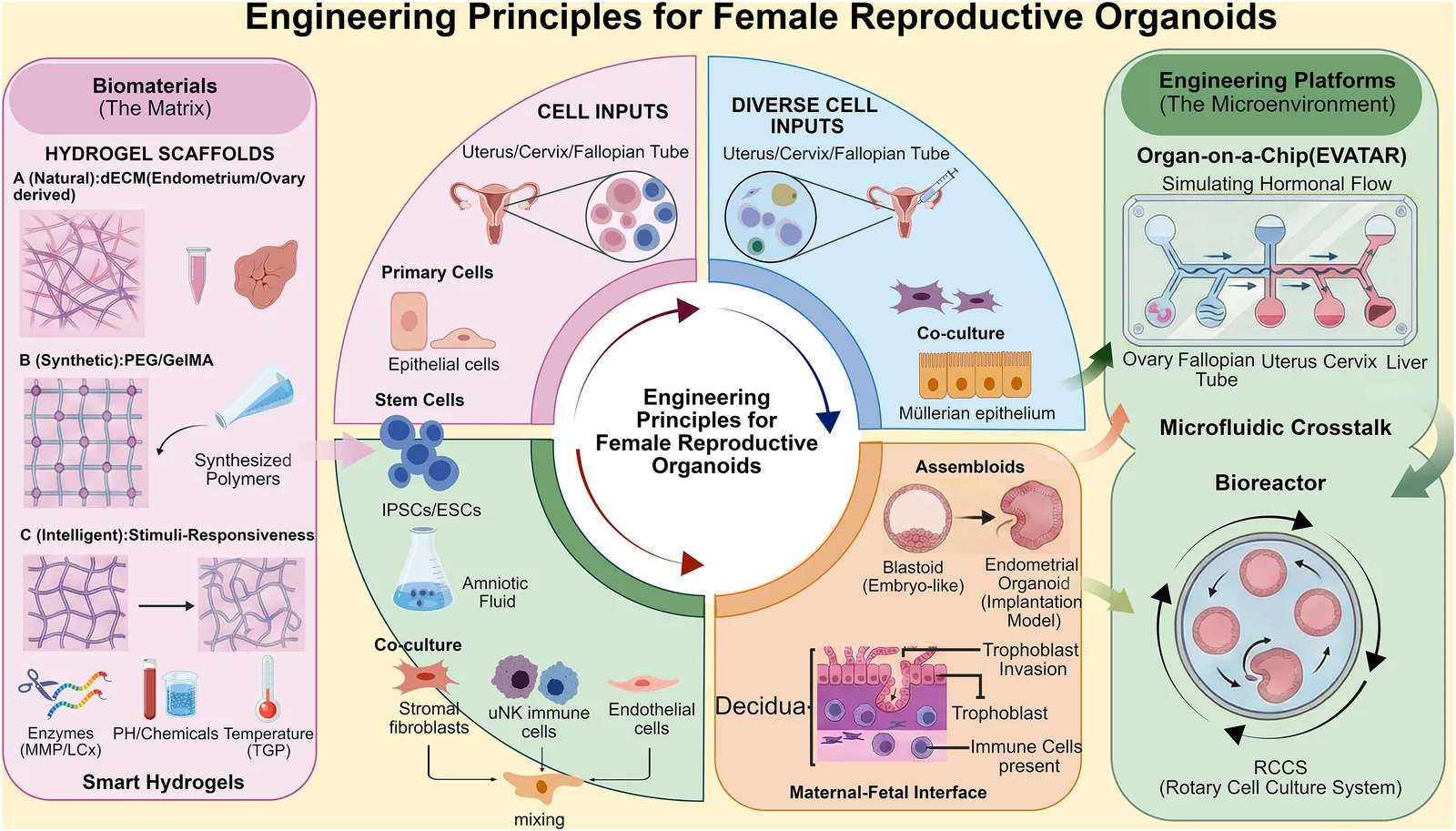
Engineering principles for female reproductive organoids. This schematic summarizes four interconnected engineering modules for constructing female reproductive organoids: biomaterials, cellular inputs, assembly strategies, and engineering platforms.

Biomaterials include natural matrices such as endometrium- or ovary-derived dECM,^13,14^ synthetic hydrogels such as PEG and GelMA,^15^ and intelligent biomaterials responsive to enzymatic, chemical, or physical cues.^16–18^

Cellular inputs include primary reproductive epithelial/stem cells, pluripotent stem cells, amniotic fluid-derived iPSC sources,^19,20^ and non-epithelial components such as stromal fibroblasts, uNK cells, and endothelial cells. Assembly strategies include assembloids for implantation modeling and maternal–fetal interface reconstruction.^21^

Engineering platforms include organ-on-a-chip systems such as EVATAR^22^ and dynamic suspension culture systems such as RCCS.^23^

### A. Biomaterials: From traditional matrices to tissue-specific scaffolds

Although widely used, traditional biomaterials show significant limitations, which have been driving the development of natural alternatives and synthetic hydrogels. Intelligent biomaterials provide novel tools for precisely mimicking physiological and pathological microenvironment through their properties of biological, chemical, and physical responsiveness.

#### 1. Traditional and natural matrices

While Matrigel is the current standard for 3D culture, its undefined composition, batch variability, and potential animal-derived-pathogens limit clinical translation.^12^ In addition, the origin of Matrigel is Engelbreth–Holm–Swarm (EHS) mouse sarcoma, possessing very different biochemical and biophysical properties from ECMs in normal or benign tissues. More detrimentally, the xenogeneic antigens within Matrigel could provoke nonspecific activation of immune cells, generating unnecessary immunological background noise.^12^ Hydrogels derived from decellularized (dECM) bovine endometrium have also been used as a scaffold for organoid construction;^13,14^ however, the xenogeneic limitations still exist. The conventional strategy attempts to subtract this baseline interference through matched experimental control designs. However, this approach assumes that matrix-derived effects behave as a linearly removable background. In practice, xenogeneic matrices such as Matrigel may influence cellular or immune responses in context-dependent and potentially non-linear ways, including synergistic priming, cascade amplification, or antagonistic masking, such that their effects cannot always be fully accounted for by simple background subtraction. Resolving this translational bottleneck demands a foundational shift toward immune-orthogonal and biologically inert or compatible biomaterials. Hydrogels derived from dECM human endometrium and ovaries are superior alternatives, providing a natural scaffold that more closely mimics the native proteome.^14,24^

#### 2. Synthetic and semi-synthetic hydrogels

PEG-based systems allow for tunable stiffness and the controlled presentation of ligands (e.g., RGD), which is critical for modeling the mechanical changes during the menstrual cycle or placental development.^15,25^ Furthermore, semi-synthetic, photo-crosslinkable materials like GelMA are highly amendable to biofabrication; when combined with sacrificial templates, they can be engineered into precise micro-cavity structures (e.g., MCO models) that mimic the spatial distribution of follicles within the native ovary.^26^ Collectively, the representative materials described earlier—such as PEG-based hydrogels, GelMA-based matrices, pH-responsive polymers, TGP, and enzyme-responsive dynamic scaffolds—demonstrate promising biocompatibility and provide tunable microenvironmental control that is particularly relevant for modeling cyclical remodeling, acidic mucosal niches, and invasion-associated matrix dynamics in the reproductive tract. These materials may facilitate the transition of organoid platforms from benchtop models toward translational and regenerative applications. In this context, biocompatible and tunable biomaterials could provide critical support for future organoid-based tissue repair strategies, such as vaginal wall reconstruction and functional restoration of the endometrium, although long-term engraftment, functional integration, and safety remain to be fully established.

#### 3. Intelligent biomaterials

Recent advances in biomaterial science have driven the evolution of biomaterials from passive structural supports to dynamic, programmable intelligent systems. Intelligent biomaterials are emerging as dynamic platforms designed to interact with biological environments precisely and purposefully and thereby realize spatiotemporal control over immunomodulation and tissue restoration.

The core feature of these bioengineered scaffolds is their ability to dynamically interact with the pathological microenvironment through programmable responses to external or endogenous stimuli. (1) Biological responsiveness: Intelligent matrices can be engineered for bidirectional, enzyme-driven dynamic remodeling. On the one hand, matrix metalloproteinase (MMP)-cleavable scaffolds are engineered to accommodate the aggressive spatial invasion of trophoblasts or cancer invasion via targeted degradation. Next-generation bio-responsive matrices integrate site-specific matrix MMP-cleavable peptides to enable cell-driven dynamic degradation and active microenvironmental remodeling.^27–29^ By precisely tuning enzymatic kinetics, the degradation rate of synthetic scaffolds can be synchronized with endogenous ECM secretion, significantly enhancing vascular integration and immunomodulatory potential. These strategies have been successfully implemented across hyaluronic acid,^28^ tetra-armed PEG,^29^ and alginate dialdehyde (ADA)^27^ platforms to reconstruct highly dynamic and functional tissue niches. On the other hand, to model pathological tissue stiffening, hydrogels (e.g., PEG-based systems) can be functionalized with specific reactive groups, such as free primary amines.^30^ Following an initial photo-polymerization that establishes the baseline structural elasticity, these biomaterials can undergo a secondary, on-demand cross-linking upon exposure to specific enzymes like lysyl oxidase (LOx).^30^ This enzymatic trigger dynamically increases the elastic modulus of the hydrogel, faithfully mimicking the progressive mechanical stiffening typical of soft tissue fibrosis. In the female reproductive tract, such LOx-responsive systems hold immense potential to recapitulate the fibrogenesis and matrix densification seen in endometriosis or uterine leiomyomas.^17,31^ (2) Chemical responsiveness: Intelligent hydrogels harness dynamic covalent chemistries^16,18^ (e.g., imine, hydrazone, boronated esters, and disulfide bonds) and reversible non-covalent interactions^32,33^ (e.g., ionic coordination) to adapt to targeted chemical cues. These covalent adaptable networks (CANs) undergo reversible topological reshuffling in response to localized pH variations, redox gradients, or specific competitive molecules. For organoid engineering, this intrinsic chemical sensitivity is dual-purpose: it empowers the matrix with biomimetic stress relaxation properties to physically accommodate aggressive cellular expansion while simultaneously enabling the on-demand, nondestructive harvesting of intact organoids through chemically triggered matrix liquefaction (e.g., utilizing targeted ion chelators or reducing agents). pH-responsive hydrogels usually composed of polymers with weakly acidic or basic moieties can dynamically adapt to the local pH fluctuation by swelling or deswelling,^34^ which may be exceptionally advantageous for engineering organoids of tissues with highly specific pH microenvironments, such as the inherently acidic vaginal tract. (3) Physical responsiveness: Some polymers can realize a reversible transition of phase triggered by a modest physical change in the environment, such as temperature and electric field.^35,36^ Utilizing a specific sol–gel transition temperature, thermo-reversible gelation polymers (TGP) function as a dynamic support system. It solidifies at physiological temperature (37 °C) to support 3D organoid formation from single stem cells but intelligently liquefies upon cooling,^37^ allowing nondestructive organoids harvesting. Magnetic fields can actuate “ferrogels” to achieve precisely timed, on-demand release of therapeutic factors. By leveraging magnetically triggered mechanical compression of biphasic scaffolds, researchers can flexibly modulate the delivery intervals of distinct cytokines, effectively guiding the local immune landscape from a proinflammatory state toward a pro-regenerative niche.^38^

### B. Cellular sources and multicellular composition

While the cyclical and biomechanical complexity of the female reproductive tract presents unique bioengineering challenges, their cellular sources do not deviate fundamentally from general organoid paradigms; both heavily depend on the expansion of tissue-resident stem cells or the directed differentiation of pluripotent stem cells (PSCs). Most models are established from epithelial stem/progenitor cells isolated from endometrial biopsies, fallopian tube fimbriae, or cervical tissues. These cells can self-renew and differentiate into multiple lineages under specific growth factor cocktails.^39–41^ Pluripotent stem cells (PSCs) offer an alternative for generating Müllerian epithelium and trophoblast lineages, particularly when primary tissues are difficult to access.^42,43^ Unlike conventional biological fluids (e.g., blood or urine), where derived cells inherently share the genetic identity of the adult host, amniotic fluid presents a unique paradigm. Although amniotic fluid is collected from the maternal gestational environment, amniotic fluid-derived cells are of fetal origin and can therefore be reprogrammed into iPSCs that preserve the fetal genetic background rather than the maternal genotype.^19,20^ This approach is extremely valuable not only in diagnosis for fetus abnormalities but also as a powerful tool to decode the early developmental molecular events governing physiological embryogenesis and the pathogenesis of congenital and genetic diseases. For instance, this approach has been applied in an early gonadal organoid construction, successfully generating human primordial germ cells-like cells and their somatic niche cells, to investigate the mechanisms of underlying impaired germline specification during early embryogenesis in Turner syndrome.^44^ This is distinct from many other patient-derived cell sources, which typically reflect the genetic background of the host individual from whom they are obtained.

Advanced models now incorporate non-epithelial components, such as stromal fibroblasts, uterine natural killer cells, and endothelial cells, to reconstruct the complex tissue niches required for implantation and pregnancy modeling.^45,46^ By bridging the gap between local tissue niches and systemic physiological networks, this co-culture strategy provides a promising platform to decipher complex host-level interactions, such as maternal–fetal immune tolerance and endocrine regulation.

### C. Assembly strategies: Assembloids and organ-level crosstalk

These strategies provide powerful tools to investigate pregnancy biology and reproductive endocrinology.

#### 1. Assembloids for implantation

Advanced assembly techniques enable the study of tissue–tissue interactions that are unique to the female system. By fusing endometrial epithelial organoids with stromal cells and embryos (or blastoids), researchers create “assembloids” to delineate the molecular roadmap of embryo implantation.^47^ During murine implantation, embryos localize within crypts extending from the antimesometrial luminal epithelium. Upon anchoring to the uterine wall with the inner cell mass (ICM) oriented toward the uterine cavity, the embryo breaches the endometrial epithelial barrier by actively orchestrating host cell apoptosis or entosis.^48,49^ In contrast to the murine model, human blastocysts orient their ICM directly toward the apical surface of the luminal epithelium, initiating a profound implantation process that culminates in the embryo becoming completely encapsulated within the endometrial stroma.^50,51^ This work demonstrates how distinct cellular modules can be spatially organized to reconstruct tissue interfaces and intercellular communication networks. Such approaches are particularly relevant for reproductive system modeling, where coordinated interactions between epithelial, stromal, and potentially immune components are essential for recapitulating physiological functions such as implantation, cyclic remodeling, and tissue regeneration.

Due to the ethical and technical constraints in acquiring early-life human biological materials, coupled with the aforementioned interspecies disparities between experimental animals and humans, such organoid technologies are of paramount importance for elucidating the processes of human reproduction and embryonic development.

#### 2. Maternal–fetal interface

The fetus functions essentially as a semi-allograft, which makes deciphering the mechanisms of maternal–fetal immune tolerance a captivating frontier.^52–54^ The breakdown of immune tolerance at the maternal–fetal interface can precipitate a spectrum of devastating obstetric complications, ranging from pre-eclampsia^55–57^ to pregnancy loss.^58,59^ Given the ethical and technical impossibility of conducting *in vivo* human studies, maternal–fetal interface organoids constructed through advanced multicellular assembly strategies to integrate placental and decidual components have emerged as advanced platforms for modeling this complex immune crosstalk. Models of the placenta involve co-culturing trophoblast organoids with decidual cells and immune cells under flow to study trophoblast invasion and the immunological mechanisms of pre-eclampsia.^60^ Unraveling the secrets of maternal–fetal tolerance not only fundamentally advances our understanding of human reproduction but also holds tremendous translational implications. Specifically, the immune-privilege strategies orchestrated by the placenta could inspire breakthrough approaches to avert rejection in allogeneic organ transplantation.^61,62^ Furthermore, malignant tumors often share similar biology with embryonic developmental programs. This phenomenon characterized by cellular dedifferentiation, the frequent re-expression of oncofetal antigens, and the orchestration of immunosuppressive niches has been widely discussed and recognized since the early stage of oncological research.^63–65^ Because of these striking “oncofetal” parallels, decoding the natural immune-privilege mechanisms at the maternal–fetal interface transcends reproductive science, for unmasking novel therapeutic vulnerabilities in cancer, potentially providing critical blueprints to overcome immune evasion and resistance in current clinical interventions, such as anti-PD-1 immunotherapies.^62^

#### 3. Bidirectional endocrine crosstalk

Integrated platforms, such as the “dual reproductive organ-on-a-chip,” allow for the study of communication between the ovary and endometrium through the diffusion of steroid hormones (E2, P4) and cytokine.^66^ As a system whose homeostasis highly relies on the harmony of hormones from hypothalamus, pituitary gland, and ovaries, reproductive endocrinology is a critical field. The dysregulation of reproductive endocrinology leads to a set of diseases such as menstrual disorders, polycystic ovarian syndrome and infertility.

#### 4. 3D bioprinting: Transitioning from stochastic to deterministic assembly

Traditional organoid generation heavily relies on the spontaneous self-organization of cells,^67^ which inevitably introduces structural heterogeneity and unpredictable spatial arrangements. 3D bioprinting fundamentally overcomes this bottleneck by enabling the deterministic, high-resolution patterning of multiple cell lineages and intelligent biomaterials (bioinks).^68–70^ As for the female reproductive tract, this biofabrication strategy is profoundly transformative for reconstructing anatomically accurate, multi-layered architectures, such as precisely separating the epithelial and stromal compartments of the endometrium or modeling the stratified squamous epithelium of the vagina. By dictating the exact spatial and topological cues, 3D bioprinting not only standardizes organoid production for high-throughput pharmacological screening but also perfectly bridges the gap between microscale cellular self-assembly and macroscale tissue engineering.

### D. Engineering platforms: Microfluidics and spatiotemporal control

The utilization of microfluidic control devices brought flow shear stress into the model, enabling hormones crosstalk between organs via circulation and providing construction technologies for specific organs.

#### 1. Microfluidic “cycle-on-a-chip” and systemic endocrine crosstalk

Advanced microphysiological platforms, such as the EVATAR system, represent a paradigm shift by fluidically linking five distinct organ units: the ovary, fallopian tube, uterus, cervix, and liver.^22,71^ Operating as an integrated “Quintet” dynamic network, this device successfully recapitulates the 28-day human menstrual cycle by faithfully mimicking the *in vivo* organ–organ crosstalk of hormonal signaling. By establishing a directional flow, downstream reproductive tissues and peripheral organs can dynamically respond to upstream ovarian hormone secretion (such as estradiol, E2), effectively capturing phase-specific molecular signatures like VEGF-A and IL-8 peaks during the follicular and luteal phases. Furthermore, the system can sustain a “pregnancy-like” state by maintaining the corpus luteum, while the strategic inclusion of hepatic microtissues allows for the inactivation and transition of hormones, systemic evaluation of drug metabolism, and reproductive toxicology. Despite its systemic complexity, the current EVATAR platform still faces significant bioengineering bottlenecks. First, as the “ovarian drive” originates from murine tissue, the system cannot spontaneously sustain a human-like 28-day cycle; instead, it relies on manual, exogenous hormone supplementation to orchestrate the transition between cycle phases. This reliance on external intervention highlights the urgent need for high-fidelity human ovarian organoids capable of autonomous endocrine regulation. Furthermore, the diverse organs within the system are typically derived from non-autologous donors with heterogeneous genetic backgrounds. This genomic disparity introduces significant confounding biological “noise” and strictly precludes the study of specific genetic perturbations (e.g., CRISPR-mediated gene silencing) on system-wide physiological outcomes, where a uniform genetic baseline across all linked tissues is indispensable.

#### 2. Organ-on-a-chip for barrier and flow

By furnishing a physiologically relevant physicochemical microenvironment, microfluidic platforms facilitate the deterministic assembly and functional differentiation of organoids across the entire reproductive tract. These systems leverage precisely controlled shear stress^72,73^ and recirculating endocrine loops to replicate the complex spatiotemporal crosstalk and mechanical cues ranging from 3D follicle expansion to mucosal barrier integrity that are essential for sustaining the dynamic hormonal exchange and tissue-level architecture^74,75^ often compromised in static cultures.^76^

#### 3. Dynamic suspension and interface-engineering

Beyond microfluidic control, macroscale suspension systems and interface-engineering strategies offer additional advantages for modeling reproductive tissues. In reproductive organoids, where multicellular architecture, epithelial polarity, and mechanical sensitivity are critical, systems such as the Rotary Cell Culture System (RCCS) leverage simulated microgravity and laminar flow to minimize detrimental shear stress while improving mass transport,^23^ thereby better supporting fragile 3D structures such as follicular or epithelial spheroids. Likewise, non-adhesive interface modification utilizing polyHEMA promotes scaffold-free self-assembly,^77,78^ which is particularly useful for generating multicellular aggregates that preserve tissue-like organization and polarity—features highly relevant for modeling ovarian surface epithelium, fallopian tube epithelium, and other mucosal reproductive tissues (Fig. 2).^79^

**FIG. 2.**
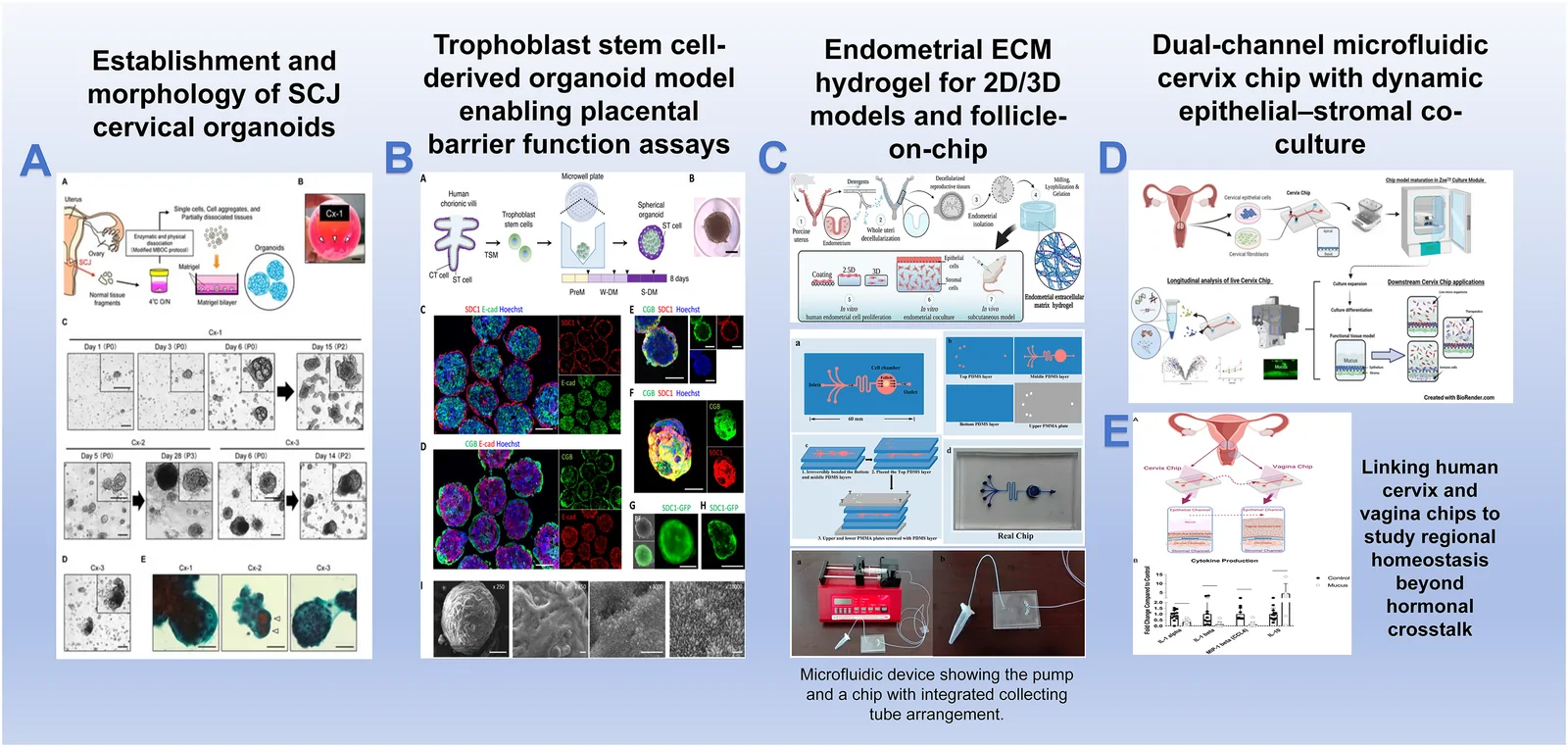
Emerging organoid and organ-on-a-chip models: Bridging engineering strategies with reproductive pathophysiology. (A) Schematic and representative morphology of squamocolumnar junction (SCJ) cervical organoids, which recapitulate key features of the native cervical transition zone.^41^ Reproduced with permission from Maru *et al.*, Cancers **12**(3), 694 (2020). Copyright 2020 Authors, licensed under CC BY 4.0. (B) Trophoblast organoid-derived placental barrier model enabling syncytiotrophoblast formation, permeability testing, and drug transport analysis.^21^ Reproduced with permission from Hori *et al.*, Nat. Commun. **15**, 962 (2024). Copyright 2024 Authors, licensed under CC BY 4.0. (C) Workflow for generating endometrium-derived extracellular matrix (dECM) hydrogels as tissue-matched scaffolds for endometrial organoid culture.^80^ Reproduced with permission from López-Martínez *et al.*, Front. Bioeng. Biotechnol. **9**, 639688 (2021). Copyright 2021 Authors, licensed under CC BY 4.0. (D) Dual-channel PDMS microfluidic cervix chip integrating epithelial and stromal compartments across a porous membrane under dynamic flow.^81^ Reproduced with permission from Izadifar and Ingber, Bio-Protoc. **15**(7), e5262 (2025). Copyright 2025 Authors, licensed under CC BY-NC 4.0. (E) Linked cervix and vagina chips connected by microfluidic interconnects for modeling lower reproductive tract homeostasis and dysbiosis-associated responses.^82^ Reproduced with permission from Gutzeit *et al.*, npj Womens Health **3**, 5 (2025). Copyright 2025 Authors, licensed under CC BY-NC-ND 4.0.

## III. ENGINEERING ORGANOIDS FOR EACH FEMALE REPRODUCTIVE ORGAN

### A. Ovaries: The germinal center of gametes and its periodic changes

The ovaries are anatomically close to fimbriae tubes, located on either side of uterus. As an important female reproductive organ, the key functions of ovary include generation of gametes and female hormones.^83^ The major cellular compositions in ovary consist of follicles, ovarian epithelium, oocytes, granulosa cells, endothelial cells, and stromal cells.^84^ During the menstrual cycle, follicles undergo dynamic changes to be released from the ovary following the luteinizing hormone surge.^85^ Constantly changing is one of the main characteristics that distinguish the ovaries from other organs, and also one of the goals and challenges in constructing ovarian organoids. Based on a 3D culture system, bioengineered ovary was created by encapsulating the follicle in the hydrogel, in which way the intercellular communication could be retained.^86–88^ However, the dynamic follicular process has not been fully recapitulated in the hydrogel, due to several reasons. To release the follicle, hydrogel-embedded systems often require matrix removal through mechanical/enzymatic methods or self-degradation strategies, which may compromise structural integrity and lack precise spatiotemporal control. In addition, conventional matrices such as MatriGel remain suboptimal for such applications because of their tumor origin, batch-to-batch variability, instability, poorly defined composition, and lack of ideal tunability.^89,90^

Biofabrication and organ-on-a-chip, combined with the utilization of different biomaterials to replace Matrigel, are promising engineering technologies, which can establish biomimetic tissues with fine-tuned architecture and biological functions to recapitulate the dynamic development process *in vivo.*^91–95^ To circumvent follicle entrapment in traditional hydrogels, a semi-opened micro-cavity ovary (MCO) was engineered using GelMA and sacrificial microspheres.^26^ This biomimetic platform facilitates the spontaneous release of mature MII oocytes, which exhibited high developmental competence, evidenced by a 25.6% IVF cleavage rate and a 0.97 transcriptomic correlation with *in vivo* counterparts. The occurrence of spontaneous parthenogenesis further validated the oocytes' meiotic maturity and intrinsic activation potential within the engineered cavities.

With the advancement of development biology and stem cells technology, 3D ovarian organoids could be generated by differentiating stem cells into germ-like cells and gametes and stomal cells through tissue engineering.^90,96^ Li *et al.* first utilized female germline stem cells (FGSCs) to generate 3D ovarian organoid models containing multiple types of ovarian lineages, such as germ, granulosa, and theca cells, which recapitulated the reproductive and endocrine functions,^97^ and also served as a novel model for reproductive toxicological test.^96^ Ovarian organoids were also established by co-culturing mouse FGSCs and ovarian somatic cells to mimic oogenesis *in vitro.*^98^ This study indicated that MitoQ could protect oocytes and granulosa cells and promote follicle growth and maturation by relieving oxidative injury and restoring mitochondrial function in oocytes. This novel *in vitro* ovarian organoid model can be used to test drug or chemical toxicity, indicating it has the potential for clinical investigation in the future.

The organ-on-a-chip technology is also developed in the field of assisted reproductive technology (ART). An optimal culture system with the microchamber array was established to promote oocyte maturation and reduce apoptosis under dynamic culture condition.^99^ The microfluidic device offers better 3D biomimetic microenvironment to the growing ovarian follicles than 2D culture systems,^95^ by allowing culturing GCs and oocytes in an interdependent manner.^100^ With the assistance of the microfluidic device, intact secondary OFs could be maintained as a functional unit for ovary study.^101^ A novel denudation-on-a-chip was created to denude oocytes from the surrounding cumulus-corona cell mass, facilitating the assessment of oocyte quality and the injection of sperm.^102^ The automation reduced variability from different operators and thereby improved standardization and ease-of-use.

The monolayer of the ovarian surface epithelium (OSE) on the surface of ovaries is widely considered to be one of the origins of malignant ovarian cancer.^100,103^ To better understand biology and tumorigenesis in ovaries, it is important to establish the *in vitro* OSE model. However, normal OSE cells usually lose some epithelial characteristics under standard 2D monolayer culture conditions due to their considerable phenotypic plasticity and exhibit both mesenchymal and epithelial features.^104,105^ An *in vitro* 3D normal OSE model was constructed from patients with bilateral salpingo oophorectomy in a RCCS and on polyHEMA-coated plastics.^106^ Both RCCS and polyHEMA OSE multicellular spheroids showed stronger expressions of fibronectin, vimentin, laminin, and AE1/AE3 than 2D cultures, which resembles features of OSE cells *in vivo* more faithfully. Del Valle *et al.* demonstrated that although 2D culture maintains high proliferation of hOSEs, the cellular features altered due to epithelial-to-mesenchymal transition (EMT), whereas 3D organoids could be passed more than four times without apparent senescence.^107^ This 3D spheroid model is a useful tool in decoding ovarian regeneration during the ovulatory cycle and injury as well as to study the earliest molecular events in ovarian oncogenesis.

OSE-derived organoids have been widely explored in the context of ovarian cancer modeling.^108,109^ Beyond their role in studying tumorigenesis,^110^ these systems also provide a valuable platform for reconstructing epithelial architecture, polarity, and microenvironmental interactions relevant to the female reproductive system. From an engineering perspective, OSE organoids enable controlled investigation of epithelial organization, matrix–cell interactions, and niche-dependent remodeling, thereby informing the design of biomaterials and culture systems for both physiological and pathological reproductive tissue models. In particular, their use in inflammatory and transformation-associated settings^111–115^ highlights how engineered organoid platforms can be adapted to model dynamic ovarian microenvironments, rather than serving solely as systems for cancer physiology.^110^

### B. Fallopian tube: An origin of ovarian cancer and transporter of sperm and fertilized eggs

Fallopian tubes (FTs) link the upper part of the uterus and the ovaries, providing the passage for the oocytes released from the ovaries. As one of the central organs of female reproduction, they are lined by epithelial cells containing secretory cells, ciliated cells, and peg cells, which produce tubular fluid and facilitate transport gametes. In addition, it has been considered as an origin of HGSC,^116–118^ which has also been verified on a fallopian tube organoid cultured on a transwell air–liquid interface expressing biomarkers for both fallopian tube and ovarian cancer.^119,120^ Thus, the complex behavior and function of fallopian tubes at the organ-level highlight the necessity to construct fallopian tube organoids as a mini *in vitro* unit to study fallopian tubes biology.

While conventional 3D culture models have successfully established human FT organoids, they traditionally rely on passive, naturally derived matrices. For instance, early approaches by Rose *et al.* encapsulated FT epithelial stem cells in Matrigel, yielding cystic organoids that mimic native tubal architecture.^121^ These static platforms provided crucial biological insights: comparative evaluations demonstrated that epithelial cells from the distal fimbria possess a higher capacity for self-renewal and organoid formation than proximal tissue. Furthermore, distal ALDH^+^ cells exhibited gene signatures characteristic of HGSC, supporting the distal FT as a highly susceptible site for malignant transformation.^121^ The essential role of the 3D matrix in maintaining stemness was further validated when transitioning cells from 2D culture to 3D Matrigel significantly increase stemness.^46^ Additionally, co-culture models incorporating FT epithelial cells, stromal cells, and human umbilical vein endothelial cells (HUVECs) revealed that organoid formation is regulated by matrix-embedded Wnt signaling pathways, which can be blocked by the Wnt inhibitor DKK.^46^

Beyond the physical scaffold, engineering the biochemical niche is also critical. Conventional defined media require costly recombinant factors (e.g., Wnt3a, RSPO1, EGF, etc.) to maintain organoid proliferation. To optimize this, a biomimetic conditioned medium derived from co-culturing epithelial, stromal, and endothelial cells at a 10:7:2 ratio was developed to simulate the native paracrine microenvironment.^122^ Although conventional media promoted faster proliferation, this engineered paracrine milieu achieved a 100% organoid formation rate and notably enhanced PAX8 and stemness gene expression, offering a cost-effective strategy for large-scale modeling.

To overcome the limitations of Matrigel, a paradigm shift toward intelligent, synthetic biomaterials is under way. Highlighting this superiority, a fully synthetic TGP was used in FT organoid establishment. TGP robustly supported organoid self-organization, preserving stemness (LGR5^+^) while enabling faithful bipotent differentiation into both secretory (PAX8^+^) and ciliated (TUBB4^+^) lineages. Crucially, its temperature-responsive nature, liquefying upon cooling, enabled enzyme-free, nondestructive organoid retrieval, providing a standardized and scalable alternative to conventional natural matrices.^37^ The biological crosstalk within the fallopian tube microenvironment is crucial for sperm functional maintenance.^123^ Gatimel *et al.* demonstrated that human fallopian tube organoids (FTOs) provide a favorable secretome that maintains superior sperm motility compared to traditional media.^124^ While this study utilized passive Matrigel scaffolds, it provides valuable insight into the essential biochemical cues that intelligent bio-instructive matrices must aim to replicate. Future designs should prioritize the integration of these organoid-derived signals into stimuli-responsive synthetic hydrogels to achieve spatiotemporal control over the sperm-oviduct interface, ultimately advancing high-fidelity ART platforms.

### C. The maternal–fetal interface: From endometrial receptivity to placental barrier

The endometrium undergoes dynamic, cyclical remodeling to establish a highly specialized microenvironment for embryo implantation and conceptus survival.^125^ Dysregulation within this delicate niche inevitably leads to severe complications, including endometriosis,^126^ infertility,^127,128^ and recurrent pregnancy loss.^129–131^ Early bioengineering efforts focused on recapitulating the foundational glandular architecture. Turco *et al.* and Fitzgerald *et al.* independently established long-term culture systems for culturing organoids from normal and decidualized human endometrium, which maintained the genetic stability and hormonal responsiveness.^11,132^ Gene expression analysis of organoids exhibited shared molecular signature with glands in original tissues. These models faithfully mimic the hypersecretory morphology characteristic of the early pregnancy decidua when stimulated by placental signals (e.g., hCG, hPL). Beyond structural resemblance, capturing the functional polarity of these tissues is critical. Utilizing a high-throughput fluid isolation approach, Simintiras *et al.* demonstrated that these organoids exhibit pronounced apical-basolateral secretory asymmetry.^133^

Solving the limitations brought by Matrigel and pushing the transition toward clinically relevant scaffolds, Jamaluddin *et al.* engineered hydrogels derived from decellularized human and bovine endometrium (dECM).^14^ By providing a tissue-matched proteomic microenvironment rich in laminin, these dECM hydrogels significantly increased the physiological fidelity of the organoids compared to conventional matrices.

Modeling the transient and highly invasive event of embryo implantation is a major challenge despite achievements on static models. To bridge the gap between isolated endometrial models and systemic reproductive physiology, Mole *et al.* engineered “CREST,” a receptive endometrial scaffold that systematically integrates luminal, glandular, and stromal compartments.^134^ This sophisticated platform supports human embryo and blastoid implantation for up to 14 days, demonstrating the PROS1-AXL signaling axis during trophoblast outgrowth. Concurrently, Song *et al.* established a 3D post-implantation assembloid by integrating human blastocysts with receptive endometrial organoids.^135^ This model successfully sustained embryogenesis through day 14 and replicated core developmental processes, including yolk sac formation and primordial germ cell (PGC) specification. This model revealed how the engineered endometrial niche actively accelerates extravillous trophoblast emergence. Collectively, these multi-lineage assembloids provided high-fidelity platforms to decode maternal–fetal crosstalk and pioneer interventions for early pregnancy loss.

Following successful implantation, the coordinated development of the human placenta is paramount for fetal survival. Because the placenta is derived from the embryonic trophectoderm, sharing the fetal genome and functioning as a semi-allograft, interspecies anatomical and immunological disparities render animal models highly inadequate for placental research.^136^ Dysfunctional placentation is the fundamental driver of severe obstetric complications, including pre-eclampsia^137–139^ and stillbirth.^140–142^

Historically, the difficulty maintaining primary cytotrophoblasts (CTBs) restricted *in vitro* investigations. However, recent bioengineering breakthroughs have bypassed these limitations. By precisely modulating the Wnt/R-spondin and TGF-β signaling pathways (e.g., utilizing CHIR99021 and A83-01), Turco *et al.*^143^ and Haider *et al.*^144^ established long-term, 3D self-renewing human trophoblast organoids that maintain bipotent differentiation capacity.

While these static organoids excel at modeling developmental trajectories, they were not able to replicate the dynamic trans-epithelial transport and hemodynamic physical cues of the maternal–fetal barrier. Simple microfluidic device limits the differentiation ratio of syncytiotrophoblast (ST) cells, which restricts the scope of longitudinal experiments.^145^ To address this, Hori *et al.* engineered a functional trophoblast barrier model utilizing a specialized column insert and a basal collagen membrane.^21^ This approach physically supports the formation of a confluent ST layer, enabling real-time transendothelial electrical resistance (TEER) measurements for tissue integrity and high-throughput drug permeability assays. Additionally, vascularization remains a critical milestone in organoid engineering. Wang *et al.* developed an engineered placental microphysiological system that integrates a vascular endothelium via a customized, pumpless biomimetic chip.^71^ This perfused “vascular niche” significantly enhances long-term trophoblast viability and extravillous differentiation and effectively models dynamic host-pathogen interactions and innate immune responses during viral infection.

### D. Cervical organoids: Niche engineering, pathogen dynamics, and mechanoregulation

The cervix serves as a critical gatekeeper within the female reproductive tract, utilizing its epithelial integrity and dynamic mucus plug as a biophysical barrier to modulate sperm passage and protect intrauterine sterility.^146^ However, the squamous-columnar junction (SCJ), which represents the interface between the endocervix and ectocervix, is a site of active squamous metaplasia. This continuous cellular turnover and the transient exposure of underlying reserve cells create a highly vulnerable anatomical niche, rendering the transformation zone (TZ), uniquely susceptible to human papillomavirus (HPV) and subsequent carcinogenesis.^147^ It is still unclear how the transition zone microenvironment is remolded during metaplasia. Organoids derived from the SCJ, including those established by Maru *et al.*, provide a robust platform that phenocopies these key features. These models maintain specific SCJ markers and exhibit dual-lineage differentiation, underscoring their physiological relevance.^41^ A central challenge in cervical engineering is the spatiotemporal control of signaling niches to sustain long-term culture. Chumduri *et al.* demonstrated that the divergent signaling requirements for the long-term culture expansion of cervical organoids effectively recapitulate the opposing Wnt signaling landscapes that govern squamocolumnar homeostasis *in vivo.*^148^ Results showed that while Wnt3A and RSPO1 are essential for endocervical organoid expansion, they are deleterious to the ectocervix, which instead necessitates forskolin (FSK) to maintain its proliferative capacity. Combined with sets of other experiments, the results indicated that cervical epithelium consists of two different cell groups regulated by opposing Wnt signaling, and a distinct boundary exists at the transition zone. By optimizing these lineage-specific cues, Hans Clevers's team also established a long-term culturing protocol of human organoids from heathy ecto- and endocervical epithelial tissues as well as cervical tumor tissues to model viral oncogenesis.^149^ These cervical organoids displayed histological and transcriptional similarity to parental tissues, sustained native secretory (MUC5AC/B^+^) and keratinization-associated signatures, and showed capacity for herpes simplex virus type 1 (HSV1). By genetically manipulating HPV16 E6E7 into healthy genomes, researchers have reconstructed phenotypes reminiscent of cervical intraepithelial neoplasia (CIN).^150^ Furthermore, integrated co-infection models in this research unmasked critical host-pathogen dynamics, revealing that *C. trachomatis* triggers hyperproliferative signals (Ki67^+^) irrespective of HPV expression.

Crucially, cervical physiology is strictly dictated by cyclical endocrine shifts, which fundamentally alter mucus rheology (e.g., ferning patterns and spinnbarkeit) to gatekeep sperm penetration.^151^ Capturing these metabolic dynamics *in vitro* represents a profound bioengineering hurdle; conventional closed-lumen 3D organoids sequester apical secretions within their core, rendering noninvasive clinical sampling methodologies impossible. To bypass this bottleneck, engineering strategies must pivot toward Air-Liquid Interface (ALI) platforms or “apical-out” organoid configurations. As demonstrated by Gurumurthy *et al.*, adapting these tissues to ALI systems exposes the apical surface, facilitating the microfluidic perfusion of cyclical hormones while potentially enabling direct, clinically relevant mucus swabbing for rheological analysis.^152^ Gurumurthy *et al.* constructed a protocol to isolate and culture epithelial stem cells from ectocervical tissues, which accommodates both the Transwell-based ALI culture system and the 3D organoid model in Matrigel.^152^ A dual-channel microfluidic “cervix chip” has been engineered by interfacing primary cervical epithelial cells with stromal cells across a porous membrane under customized dynamic flow. This platform faithfully recapitulates the human cervical barrier and structural functions, providing a robust preclinical model for studying conditions ranging from viral oncogenesis to preterm labor-associated dysfunction.^81^

### E. Vagina: Microbiota and immunity

Engineering high-fidelity vaginal microphysiological systems necessitates reconstructing a dynamically estrogen (E2)-responsive, multistratified biophysical barrier. A critical design parameter is the deterministic control of the adult stem cell niche. Recent murine models demonstrate that the *in vitro* assembly of basal, suprabasal, and cornified apical layers relies on defining a precise biochemical blueprint.^153^ Specifically, canonical Wnt/β-catenin signaling and E2 act as essential bio-cues driving multipotent K14^+^ and Axin2^+^ progenitors toward targeted lineage specification, yielding a differentiated suprabasal layer by day 7 and a mucin-secreting lumen by day 21.^154^

Beyond structural mimicry, advancing these platforms requires the modular integration of immune components. By co-culturing vaginal epithelial organoids with CD8 T cells, researchers have engineered immune-competent reductionist systems capable of generating tissue-resident memory T cells (TRMs) *in vitro*. This controlled architecture supports HSV-1 and HSV-2 replication while isolating critical variables, enabling precise quantification of spatiotemporal epithelial-immune crosstalk during viral infections.^155^

Host–microbe interactions in the vaginal tract play a pivotal role in modulation of vaginal health and disease.^156,157^ A persistent bioengineering bottleneck in modeling the host-microbiome axis is the rapid bacterial overgrowth and epithelial apoptosis inherent to static co-cultures. Microfluidic organ-on-a-chip devices circumvent these limitations via dynamic fluidic control,^158^ providing dynamic fluidic shear forces to physically mimic physiological luminal clearance and actively washing away excess microbes to prevent toxicity. In another study, this design sustained living microbiota in contact with human intestinal epithelium for at least five days.^159^ To construct a resilient mucosal interface capable of anchoring these living microbes, based on this strategy, Mahajan *et al.* developed a dual-channel “vagina-on-a-chip” integrating primary human epithelial cells in the apical channel and uterine fibroblasts in the basal channel.^160^ Driven by intermittent perfusion, this synergistic crosstalk spontaneously constructs a robust, 75–90 *μ*m thick stratified epithelium. This high-fidelity architecture sustains a strict permeability barrier for 15 days, expressing pan-layer structural markers (e.g., cytokeratins 5/14/13/15, occludin-1, and desmoglein-1/3), thereby providing a structurally competent niche to safely co-culture vaginal microbiota without compromising host tissue viability. Leveraging this structurally competent niche to map host-microbiome symbiosis, the chip sustains living microbiota: Lactobacillus suppresses proinflammatory cytokines, whereas Gardnerella vaginalis induces barrier disruption, elevated pH, and bacterial vaginosis (BV)-like inflammation. Furthermore, dose-dependent transcriptomic sensitivity to cyclical β-estradiol perturbations (e.g., dynamic ESR1 and tight junction regulation) validates the system's functional fidelity. Consequently, this scalable microfluidic platform enables robust vaginal microecology decoding and live biotherapeutic product (LBP) evaluation.

### F. Modeling bidirectional crosstalk: Multi-organ microphysiology

Individual organoid models, while informative, inherently lack the systemic endocrine feedback loops essential for female reproductive physiology. For instance, the ovary and endometrium are governed by strict bidirectional regulation:^161^ the ovary dictates endometrial receptivity via estrogen (E2) and progesterone (P4),^162^ while the receptive endometrium synthesizes critical local factors, such as prostaglandin E2 (PGE2), which retroactively facilitate follicular rupture and ovulatory functions.^163,164^ To capture this dynamic interplay, Park *et al.* engineered an innovative “dual reproductive organ-on-a-chip.”^66^ This compartmentalized microfluidic device fluidically interconnects endometrial and ovarian modules, enabling the bidirectional diffusion of E2, P4, and PGE2. By successfully recapitulating this interdependent endocrine crosstalk, the platform demonstrated significantly enhanced metabolic viability and physiological fidelity compared to isolated cultures. Furthermore, by integrating a SERPINB2 luciferase reporter system into the engineered tissue, this dual-organ chip serves as a highly robust, real-time biosensor for screening the reproductive toxicity of hazardous substances, showcasing the immense translational potential of multi-organ microphysiology.

Beyond hormonal crosstalk, recent advancements have enabled the physical linkage of human cervix and vagina chips to study regional homeostasis.^82^ This integrated system demonstrates that cervical mucus acts as a critical protective barrier, significantly reducing inflammation and epithelial damage induced by a dysbiotic microbiome, such as those associated with bacterial vaginosis (BV). By mimicking the inter-organ flow of cervicovaginal fluid, these linked platforms unmask the essential physiological synergy required to maintain reproductive health.

Here, we are presenting a summary of common engineering methods for organoids and organ-on-a-chips in each female reproductive organ (Table I).

**TABLE I.** Engineering strategies for female reproductive organoids: applications, advantages, and limitations.

Engineering strategy	Applied organoids	Advantages	Limitations	Refs.
Microfluidic chip/organ-on-a-chip	Ovary, fallopian tube, endometrium, placenta cervix, vagina, multiorgan	– Provides dynamic flow and shear stress, mimicking *in vivo* microenvironment	– Complex device fabrication	22, 66, 71, 72, 74, 76, 81, 82, 94, 95, 99, 101, 102, 145, 146, 158–160
		– Enables real-time monitoring and automation	– Limited standardization	
		– Supports inter-organ endocrine crosstalk	– Long-term cell viability challenging	
		– Allows co-culture with microbes	– Difficult to scale up	
Air–liquid interface	Cervix epithelium, fallopian tube epithelium	– Promotes epithelial polarization and apical access for sampling	– Difficult to maintain long-term	119, 120, 152
		– Better mimics mucosal barrier function	– Apical exposure may cause drying	
		– Provide microgravity, promotes 3D aggregation	– Not applicable to all organs	
			– Special equipment required	
Rotary cell culture system	Ovarian surface epithelium	– Enhances ECM expression, reduces EMT	– Incompatible with real-time imaging	23, 106
		– Preserves *in vivo*-like phenotype	– Not suitable for static-dependent cells	
		– Recapitulates paracrine signaling and cell–cell interactions		
Co-culture systems	Fallopian tube, endometrium, placenta, vagina, cervix	– Enhances organoid maturation and function	– Complexity in media optimization	45–47, 60, 122, 150, 155
		– Enables study of host–microbe/immune dynamics	– Risk of overgrowth of one cell type	
			– Difficult to deconvolve individual contributions	
Synthetic/smart biomaterials	Ovary, fallopian tube, endometrium, general 4D matrices	– Tunable mechanical and degradation properties	– Biocompatibility validation needed	12, 14–16, 18, 25–27, 29, 30, 37
		– Enzyme-free retrieval–tissue matched biochemical cues	– Complex synthesis	
		– Programmable release of factors	– Regulatory hurdles for clinical translation	
Stem cell/organoid co-culture	Ovary, endometrial organoids from iPSCs, trophoblast organoids	– Enables generation of multiple lineages from stem cells	– Lineage-specific differentiation efficiency varies	9, 19–21, 39, 41–44, 90, 96, 97, 143, 144
		– Allow genetic manipulation and disease modeling	– Immaturity of some iPSC-derived organoids	
		– Recapitulates developmental processes	– Ethical concerns for certain sources	
Multi-organ/assembloid platforms	Ovary-endometrium dual chip, cervix-vagina linked chip, post-implantation assembloid, CREST scaffold	– Models systemic endocrine loops and inter-organ communication		
		– Reveals emergent properties	– Increased complexity in design and analysis	47, 66, 82, 134, 135
		– Enables study of implantation and early development	– Difficult to maintain multiple cell types long-term	
			– High cost	
Future directions				
4D bio-instructive matrices	iPSC-derived reproductive organoids	– Time as fourth dimension: dynamic tuning of cues	– Still in early development	16, 18, 30, 166, 179
		– Can drive terminal maturation and standardize differentiation	– Requires precise control of multiple parameters	
Isogenic multi-organ platforms	Patient-specific ovary, fallopian tube, endometrium, immune cells from same somatic source	– Eliminates immunogenetic confounding	– Technically challenging to derive all lineages from one source	6, 165–167, 169
		– Enables personalized maternal–fetal immune tolerance studies	– Long culture times	
Cybernetic/closed-loop systems	Any organoid integrated with biosensors + machine learning	– Real-time feedback via FRET sensors	– Requires advanced sensor integration	27, 28, 38, 167, 184
		– Autonomous microenvironment control	– Data interpretation complex	
		– Predictive modeling of drug responses	– High initial investment	
Region specific organoids	Fallopian tube, cervix	– Recapitulates anatomical zonation using spatial transcriptomics blueprints	– Requires high-solution biofabrication	148, 165, 190, 191
		– Uncovers regional disease susceptibility	– Maintaining gradients long-term is difficult	
Electro-active contractile organoids	Myometrium/smooth muscle-integrated organoids	– Conductive scaffolds guide anisotropic alignment	– Materials biocompatibility and conductivity need optimization	191–196
		– Enables electrical pacing to study coordinated contraction	– Integration with epithelial compartments challenging	
		– Models dystocia and drug effects		

## IV. CHALLENGES AND FUTURE PERSPECTIVES

While we have gained plenty of valuable insights into female reproductive system on organoids and organ-on-a-chip, current models are insufficient to meet the rigorous demands of medical research. To address this translational deficit, we are delineating the imperatives for medical research, summarizing the prevailing constraints of existing models and proposing potential strategic engineering frameworks to enhance the physiological relevance of these models (Fig. 3).

**FIG. 3.**
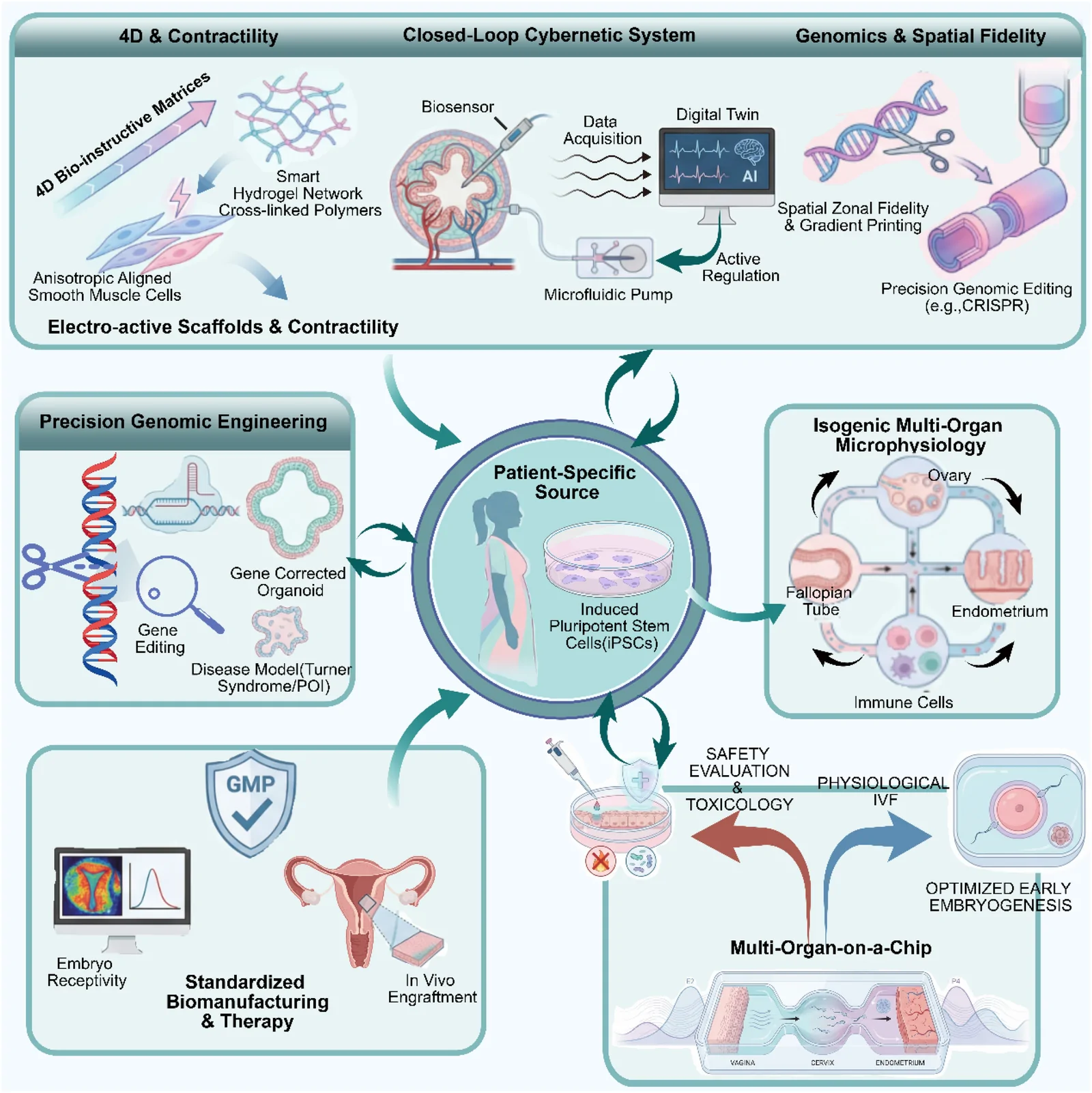
Engineering next-generation reproductive organoids: a roadmap from patient-specific cells to clinical applications. This schematic summarizes a translational roadmap from patient-specific somatic cells/iPSCs to clinically relevant reproductive organoid platforms. Three major engineering directions are highlighted: (i) precision genomic engineering and spatial fidelity,^165^ (ii) 4D bio-instructive matrices and contractile scaffolds,^166^ and (iii) closed-loop cybernetic systems integrating biosensors and digital twin-guided feedback regulation.^167^ These strategies converge to support isogenic multi-organ microphysiology platforms and, together with standardized biomanufacturing, may enable applications in optimized IVF, regenerative therapy, and high-fidelity toxicology and safety evaluation.

### A. 4D Bio-instructive matrices to overcome the “Maturation Bottleneck”

While current 3D organoids faithfully recapitulate early developmental stages, their final maturation levels often show significant variability. Specifically, distinct iPSC lines can display highly heterogeneous differentiation efficiencies when generating the same organoid type; conversely, a single iPSC line may exhibit inconsistent maturation states when directed toward distinct reproductive lineage.^169^ Additionally, organoids derived from ASCs in primary tissue, usually present a higher level of maturation, sharing more similarity with adult tissues; however, organoids derived from iPSC are more like fetal tissues.^179^ This cell-line and lineage-specific inconsistency constitutively hinder the modeling of adult-onset reproductive pathologies. Future engineering efforts must change from static, “one-size-fits-all” 3D scaffolds to 4D bio-instructive materials, as the fourth dimension is *time.*^167^ Material science shows a possibility to provide a solution to help increase the maturation level. By integrating stimuli-responsive hydrogels characterized by programmable degradation kinetics, dynamic cross-linking, tunable stress relaxation profiles, and cytokines on-demand releasing, researchers will be provided with sophisticated options of intelligent biomaterials.^180^ This high-resolution tunability allows for the dynamic modulation of physicochemical parameters perfectly tailored to the unique developmental kinetics and intrinsic biases of specific cell lines or target organs. By applying these customized spatiotemporal biomechanical cues, 4D matrices can artificially drive the terminal maturation and standardize the differentiation outcomes of iPSC-derived reproductive organoids.

### B. Patient-specific isogenic multi-organ microphysiology

Current “Cycle-on-a-Chip” platforms, which utilize non-autologous tissues, introduce severe immunogenetic confounding factors.^22^ It is obviously necessary to develop fully isogenic multi-organ platforms, where the ovary, fallopian tube, endometrium, and local immune compartments are co-differentiated from a single patient's somatic cells. This strictly controlled isogenic baseline is critical for conducting high-fidelity genetic investigations and decoding maternal–fetal immune tolerance. Additionally fascinating, the meaning of modeling this precise immunological crosstalk is far beyond reproductive biology; it profoundly deepens our understanding of systemic immune regulation, offering fundamental and transformative insights into autoimmune disorders, allograft rejection, and tolerance in organ transplantation^6,170^ and ultimately revealing the complex oncofetal mechanisms.^171–173^

### C. Vascularization as a critical bottleneck in reproductive organoid engineering

Vascularization is a critical bottleneck in advancing reproductive organoids toward greater physiological relevance and translational utility. Beyond oxygen and nutrient supply,^174^ vascular networks are essential for tissue remodeling,^175^ biochemical gradients,^176^ and long-term structural stability,^177^ particularly in reproductive tissues such as the endometrium and placenta.^178,181^ Recent studies have begun to demonstrate the feasibility of vascularized reproductive microphysiological systems. Examples include a placental organoid microphysiological system incorporating vascular endothelium and vascularized endometrial platforms integrating epithelial organoids, stromal cells, and endothelial cells. However, achieving stable perfusable vasculature, appropriate spatial organization, and long-term coupling with cyclic hormonal remodeling remains challenging.^178,181,182^

### D. Immune integration and co-culture in reproductive organoid systems

The integration of immune components represents another critical frontier in reproductive organoid engineering. The female reproductive tract relies on tightly regulated immune–epithelial–stromal interactions for implantation, mucosal defense, and maternal–fetal tolerance.^45,183^

Incorporating immune cells through co-culture strategies may therefore improve the physiological relevance of reproductive models. Representative examples include endometrial organoid systems used to study epithelial–immune interactions in infection,^45^ matched trophoblast and decidua organoids used to dissect antiviral and immunomodulatory signaling at the maternal–fetal interface,^183^ and vaginal epithelial organoid–CD8 T cell co-culture systems that support resident memory T cell differentiation.^155^ However, maintaining immune cell viability, controlling activation states, and reproducing context-appropriate immune interactions remain major challenges.^184^

### E. Closed-loop microenvironment design: Integrating biosensors and intelligent biomaterials

One of the ultimate goals in organoid engineering transcends passive monitoring. Active, real-time feedback regulation is highly demanded. Future platforms are expected to seamlessly integrate in-line, nondestructive biosensors with stimuli-responsive intelligent biomaterials to create fully autonomous, “cybernetic” culture systems.^168^ For instance, a FRET-based peptide reporter covalently anchored within intelligent matrices has been developed, where enzymatic cleavage separates the fluorophore from the quencher to trigger a quantifiable “turn-on” signal indicative of localized proteolytic activity.^27^ In a closed-loop design, these indictive signals provide sensory input for computational models that decode localized biochemical changes to generate actionable feedback. Specific signals can trigger programmed structural or chemical transformations, such as scaffold degradation,^28^ real-time stress relaxation to accommodate trophoblast invasion, or initiating the on-demand release of immunomodulatory factors/cyclical hormones (E2, P4) to realize regulation.^38^ Combining data acquired from this platform with machine learning algorithms, researchers can establish predictive computational models, often conceptualized as “Digital Twins” of the female reproductive tract or “virtual organoids.”

Despite its strong conceptual appeal, the implementation of digital twin systems in reproductive organoid platforms faces several critical challenges. First, the integration of multi-scale and multi-modal data, including molecular, cellular, and tissue-level information, remains technically demanding.^185^ Second, the lack of robust real-time sensing technologies limits the ability to dynamically capture system states for model updating.^186,187^ Third, model validation is inherently difficult due to biological variability and the absence of gold-standard benchmarks.^187,188^ Finally, inter-individual heterogeneity further complicates the development of predictive and generalizable models.^185,188^ Addressing these challenges will be essential for translating digital twin concepts into practical and reliable tools.

### F. Standardized biomanufacturing and clinical translation: From diagnostics to regenerative therapeutics

To successfully transition organoids from lab models to direct clinical applications, establishing standardized biomanufacturing protocols is significant. This requires a full adoption of GMP-compliant, xeno-free intelligent biomaterials coupled with strict quality control (QC) metrics to eliminate structural variability and guarantee absolute *in vivo* safety.^169^ In the near term, this standardized platform will revolutionize precision diagnostics, establishing patient-derived endometrial organoids as high-fidelity clinical tools to definitively assess personalized embryo receptivity prior to IVF. Beyond diagnostics, the field is advancing toward *in vitro* therapeutic platforms where organoids serve as “physiological feeder systems” to optimize gamete handling. For instance, acellular microfluidic platforms using hyaluronic acid have helped with the precise biomechanical quantification of sperm locomotion within a biomimetic cervical mucus model.^146^ This approach may address a critical clinical need for healthy sperm selection in ART. These integrated platforms offer transformative value as noninvasive diagnostic tools to evaluate personalized sperm-mucus compatibility, ultimately enhancing the precision and success rates of clinical fertility interventions. In the future, integrated human “cervix-on-a-chip” and “fallopian tube” secretomes have the potential to facilitate *in vitro* sperm capacitation and acrosome integrity.^81,124^

Beyond diagnostic screening, organoid-based regenerative medicine has always been a goal that this field pursues. Engineered multicellular constructs, such as transplantable ovaries organoids or endometrial organoid patches, have brought exciting hope for direct *in vivo* engraftment.^24^ By safely restoring systemic endocrine homeostasis and regenerating structural tissue integrity, these implantable, clinical-grade organoid systems could fundamentally cure irreversible reproductive pathologies, such as premature ovarian insufficiency or Asherman's syndrome, thereby redefining the future of fertility restoration technologies.

### G. Precision genomic and chromosome engineering for rare reproductive pathologies

While most of the current organoid models focus on acquired diseases, modeling congenital abnormalities and rare reproductive disorders, such as Turner syndrome (45,X),^44^ Mayer–Rokitansky–Küster–Hauser (MRKH) syndrome,^189^ and genetic primary ovarian insufficiency, remains a major challenge. Advanced genomic and chromosome-level editing technologies (e.g., CRISPR-Cas9, prime editing, and targeted chromosome elimination/duplication) might be useful tools to address this challenge.^190^ By establishing isogenic organoid models, researchers can elegantly uncouple specific monogenic or chromosomal aberrations from patient genetic background noise. With these engineered platforms, we will be able to uncover the molecular etiology of early gonadal dysgenesis and develop targeted gene therapies and fertility restoration strategies for patients with rare genetic disorders.

### H. Advancing “spatial fidelity”: Engineering region-specific micro-niches

Although the organoid models we are using today successfully recapitulate generic organ functions, they fundamentally lack anatomical “resolution.” Organs are not *in vivo* homogenous cellular masses; they exhibit significant spatial heterogeneity and zonation. Within the female reproductive tract, these subtle microenvironmental gradients strictly dictate disease tropism. For example, the distal fimbriae of the fallopian tube are uniquely susceptible to HGSC initiation, whereas the proximal isthmus is not;^118,191^ similarly, the cervical SCJ represents a vulnerable area to viral oncogenesis. However, conventional organoid protocols frequently yield “bulk” or pan-tissue identities that completely mask these vital zonal differences.

Achieving spatial fidelity will be extremely meaningful, which has been happening in brain organoids development. An omics-directed approach might be helpful. First, integrating single-cell multi-omics and spatial transcriptomics is required to generate a mathematical landscape of the precise spatiotemporal morphogen gradients (e.g., Wnt, BMP, retinoic acid) that define distinct anatomical sub-regions.^166^ Subsequently, bioengineering must translate these multi-omic blueprints into physical reality. By applying microfluidic control devices to establish continuous morphogen gradients or utilizing high-resolution 3D bioprinting, future platforms could construct spatially patterned organoids, seamlessly transitioning from proximal to distal phenotypes within a continuous chip or a subarea organoid.^192^ Ultimately, these high-resolution, region-specific platforms will not merely replicate *in vivo* zonation but serve as powerful discovery engines to reveal the complex microenvironmental factors underlying regional disease vulnerabilities.

### I. Engineering functional contractility: Decoding “contractile vectors” via electro-active intelligent scaffolding

A critical limitation of current female reproductive organoids is their “epithelial-centric” bias. Most existing models are, in anatomical and histological reality, “tissue-oids,” simple stratified, or tubular epithelia that lack the structural and functional complexity of true organs. The human reproductive tract is fundamentally a mechanosensitive, contractile system; however, the complete absence of a functional mesenchymal-muscular framework hinders the study of pathologies driven by mechanical dysfunction.^193^

A perfect example is dystocia (dysfunctional labor), which often stems from incoordinate uterine activity where the contractile vectors of the myometrium fail to synchronize.^194,195^ The human myometrium is not a homogenous muscle mass but a sophisticated, anisotropic architecture of longitudinal, circular, and oblique fibers. Any disruption in this spatial alignment or electrical coupling leads to misdirected forces that hinder fetal descent.

The integration of electro-active and stimuli-responsive biomaterials into multi-tissue assembly is the key to solve this problem. Rather than utilizing static scaffolds, future platforms will leverage conductive 4D-printed matrices capable of reacting to the change of electric fields and modulating the electrical syncytium of the engineered tissue.^195,196^ These intelligent materials are expected to actively guide the anisotropic alignment of iPSC-derived smooth muscle cells during development. During functional phases, the conductive network within the bio-scaffold can assist synchronous electrical coupling, effectively “pacing” the organoid to generate coordinated contractile waves.^196^ By “wrapping” epithelial lumens within these bio-instructive, electro-responsive sheaths, we can create functional models capable of mimicing the rhythmic, directional peristalsis intrinsic to human reproduction. Such intelligent-material-enabled systems will become powerful tools for unmasking the biophysical origins of labor complications and evaluating next-generation tocolytic or oxytocic therapies.

### J. From reductionism to holistic integration: Engineering high-dimensional physiological systems

The next paradigm shift in reproductive bioengineering needs a transition from reductionist deconstruction where isolated factors such as pH, specific cell types, or extracellular matrices are studied separately, toward holistic physiological integration. Current microphysiological systems (MPS) often function as “factor-isolated” models that only possess some properties of the native environment. Future roadmaps must prioritize the engineering of increasingly complex, multi-parametric platforms capable of recapturing the spatiotemporal hallmarks of human physiology, most notably endocrine periodicity and multi-lineage dynamic synergy, while also capturing the dynamic male–female interface.

Another function needed for these systems is the “gamete-compatibility” of material to ensure that intelligent matrices assist, rather than hinder, sperm motility and acrosome integrity. Crucially, by linking male reproductive cues with integrated cervix-vagina-endometrium axes, these systems will enable the development of “Physiological IVF” platforms. Unlike traditional static cultures, these microfluidic environments can recapitulate the sequential biochemical and biophysical environment and function of the female tract, ranging from sperm chemotaxis and selection to fertilization and providing a more authentic and optimized milieu for early embryogenesis.

Beyond medical applications, these integrative platforms will revolutionize the safety evaluation of reproductive health products, such as personal lubricants and barrier contraceptives. While a mere Vagina Chip provides basic toxicological data, it fails to take a protective role of cervical mucus, which has been shown to modulate vaginal dysbiosis and shield the epithelium from inflammatory damage.^82^ By engineering a linked cervix-vagina axis that incorporates multi-organ secretomes (e.g., cervicovaginal fluid), researchers can assess how exogenous chemicals interact with the complex homeostatic environment in the reproductive tract, ensuring that consumer products maintain mucosal integrity and gamete compatibility more precisely.^146^

## Data Availability

Data sharing is not applicable to this article as no new data were created or analyzed in this study.
